# A hybrid gene selection approach to create the S1500+ targeted gene sets for use in high-throughput transcriptomics

**DOI:** 10.1371/journal.pone.0191105

**Published:** 2018-02-20

**Authors:** Deepak Mav, Ruchir R. Shah, Brian E. Howard, Scott S. Auerbach, Pierre R. Bushel, Jennifer B. Collins, David L. Gerhold, Richard S. Judson, Agnes L. Karmaus, Elizabeth A. Maull, Donna L. Mendrick, B. Alex Merrick, Nisha S. Sipes, Daniel Svoboda, Richard S. Paules

**Affiliations:** 1 SciOme LLC, Research Triangle Park, North Carolina, United States of America; 2 Division of the National Toxicology Program, National Institute of Environmental Health Sciences, National Institutes of Health, Research Triangle Park, North Carolina, United States of America; 3 Division of Intramural Research, National Institute of Environmental Health Sciences, National Institutes of Health, Research Triangle Park, North Carolina, United States of America; 4 Division of Extramural Research and Training, National Institute of Environmental Health Sciences, National Institutes of Health, Research Triangle Park, North Carolina, United States of America; 5 National Center for Advancing Translational Sciences, National Institutes of Health, Rockville, Maryland, United States of America; 6 National Center for Computational Toxicology, Office of Research and Development, U.S. Environmental Protection Agency, Research Triangle Park, North Carolina, United States of America; 7 National Center for Toxicological Research, U.S. Food and Drug Administration, Jefferson, Arkansas, United States of America; Oregon State University, UNITED STATES

## Abstract

Changes in gene expression can help reveal the mechanisms of disease processes and the mode of action for toxicities and adverse effects on cellular responses induced by exposures to chemicals, drugs and environment agents. The U.S. Tox21 Federal collaboration, which currently quantifies the biological effects of nearly 10,000 chemicals via quantitative high-throughput screening(qHTS) in *in vitro* model systems, is now making an effort to incorporate gene expression profiling into the existing battery of assays. Whole transcriptome analyses performed on large numbers of samples using microarrays or RNA-Seq is currently cost-prohibitive. Accordingly, the Tox21 Program is pursuing a high-throughput transcriptomics (HTT) method that focuses on the targeted detection of gene expression for a carefully selected subset of the transcriptome that potentially can reduce the cost by a factor of 10-fold, allowing for the analysis of larger numbers of samples. To identify the optimal transcriptome subset, genes were sought that are (1) representative of the highly diverse biological space, (2) capable of serving as a proxy for expression changes in unmeasured genes, and (3) sufficient to provide coverage of well described biological pathways. A hybrid method for gene selection is presented herein that combines data-driven and knowledge-driven concepts into one cohesive method. Our approach is modular, applicable to any species, and facilitates a robust, quantitative evaluation of performance. In particular, we were able to perform gene selection such that the resulting set of “sentinel genes” adequately represents all known canonical pathways from Molecular Signature Database (MSigDB v4.0) and can be used to infer expression changes for the remainder of the transcriptome. The resulting computational model allowed us to choose a purely data-driven subset of 1500 sentinel genes, referred to as the S1500 set, which was then augmented using a knowledge-driven selection of additional genes to create the final S1500+ gene set. Our results indicate that the sentinel genes selected can be used to accurately predict pathway perturbations and biological relationships for samples under study.

## Introduction

Several studies have demonstrated the utility of harnessing the power of transcriptomic signatures for understanding normal biology, elucidating mechanistic processes in disease and toxicity and identifying predictive signatures of biological alterations. The Toxicology in the 21st Century, or Tox21, program is a U.S. government multiagency collaborative effort among two partners from the National Institutes of Health (NIH), the National Toxicology Program (NTP) of the National Institute of Environmental Health Sciences (NIEHS) and the National Center for Advancing Translational Sciences (NCATS), together with the U.S. Environmental Protection Agency (EPA) National Center for Computational Toxicology (NCCT) and the U.S. Food and Drug Administration (FDA) [1]–[4] with the objective to improve the assessment of chemical hazards by generating high quality, target-specific, mechanism-based biological information largely obtained using high-throughput and high-content *in vitro* assays, with an aim towards reducing, refining, and, to a large extent replacing the use of animals in toxicity testing. More specifically, the goals of the program are to identify patterns of chemically induced biological responses to characterize toxicity and disease pathways, prioritize compounds for more extensive toxicological evaluation, and develop models predictive of adverse health effects in humans. Tox21 Phase I, which was essentially a proof of principle effort, is completed and Phase II, an expanded compound screening effort, is in progress. In Tox21 Phase II, approximately 10,000 compounds are being screened across a focused set of nuclear receptor and stress response pathway targeted tests [4]. In addition to providing valuable insight into characteristics associated with the selected chemicals, Tox21 Phase II results have provided significant lessons learned and have helped in the identification of the limitations of the initial high-throughput screening (HTS) assays. These include, for example, incomplete pathway coverage (i.e., a focus mainly on nuclear receptor and stress response pathways), the focus on single compounds and acute exposure scenarios, and a lack of biological complexity (e.g., the use of reporter gene assays using transformed cell lines with limited capability for xenobiotic metabolism). To address some of these limitations, the Tox21 consortium is now seeking to incorporate more physiologically relevant cell types (e.g., human hepatocytes, both primary and spheroid HepaRG cells, human ES and iPSC-differentiated cell populations) and simple model organisms (e.g., zebrafish), coupled with high-content screening and high-throughput transcriptomics platforms to assess chemical toxicity potential. Ultimately, the hope is that “omic” (whole system) approaches, such as whole genome transcriptomics, can help link perturbations, such as chemical exposures, with alterations in biological processes that result in toxicity and/or disease. However, full transcriptome experiments using microarrays or RNA-Seq remain cost prohibitive when the number of samples is very large. For this reason, the Tox21 program is exploring alternative approaches and platforms that allow for targeted gene expression using a carefully selected subset of the transcriptome.

It has been postulated that the high degree of co-expression naturally occurring in the transcriptome of living systems can be used to infer transcriptional profiles of genes without measuring every individual gene in the transcriptome [5]. This rationale led Golub and colleagues in developing and utilizing the L1000 platform that is currently being used as part of the NIH Common Fund sponsored Library of Integrated Network-based Cellular Signatures (LINCS) program [6,7]. Similarly, to use transcriptional profiling for toxicological hazard assessment, the Tox21 program recently embarked upon selection of toxicologically relevant “sentinel” genes to be used for screening purposes. An appropriate set of representative genes for the sentinel gene set must provide maximal information useful for understanding the mechanisms of adverse effects from chemical exposures and provide health scientists and regulators with critical information to assist in dose-dependent and time-dependent hazard identification. The goal was to select genes that cover all known biological pathways (per MSigDB v4.0) and to ensure that genes, gene sets, and pathways of special interest to toxicological assessment are adequately represented in the resulting sentinel gene set. Additionally, the goal was to construct a gene set that embodies the biological diversity present in the publicly available transcriptional data, and includes genes likely to be key predictors of the expression profiles of other genes.

To achieve these objectives, we developed a novel, modular bioinformatics approach which is a hybrid of both data-driven and knowledge-driven methods. The data-driven component results from a characterization of the gene expression diversity and co-expression that is represented in publicly available transcriptomics datasets. Meanwhile, the knowledge-driven component included soliciting, compiling, and integrating genes nominated by scientists with expert knowledge in toxicology and other related fields. The nominated genes represent pathologically and toxicologically relevant genes known to be of value in characterization of disease and toxicity processes. Scores from the data-driven component were used to generate an initial set of 1500 genes, which was then optimized using an algorithm that performed replacement of genes to ensure maximal pathway coverage. The resulting gene set was then enhanced with the knowledge-driven gene list, thus creating the final “S1500+” sentinel gene list.

## Methods

### Gene expression data

For our gene selection approach to robustly reflect a variety of biological settings and experimental conditions, we first required a large amount of whole genome expression data from a diverse set of experiments. Since gene expression data originating from different platforms may contain significant platform-specific biases [8,9], we chose to utilize data from a single platform type: Affymetrix Human Whole Genome Microarrays (HG-U133plus2). This platform represents the largest percentage of the publicly available human microarray data found in the Gene Expression Omnibus (GEO) repository [10]. All HG-U133plus2 datasets publicly available from the NCBI GEO website were downloaded on February 3, 2014. This large collection embodies extensive transcriptomic diversity and consists of more than 200 different organ, tissue and cell types along with various carcinomas. This large collection served as our “training set” and included 91,491 samples from 3,339 GEO series (studies). In addition to downloading the raw expression data (CEL files), we also simultaneously obtained the corresponding sample annotations from GEO. Manual curation was used to match each test sample to its control sample(s) using either the experimental design details from sample annotations or any other relevant citations given by GEO for the associated data series (see Page 1 in S1 File, for details on manual curation of GEO data). Samples were discarded if no reliable control/treatment pairing could be discerned from the provided data or if the corresponding CEL files are unavailable, resulting in a final curated dataset containing 77,985 samples.

Raw signal from Affymetrix CEL files was extracted using the Affymetrix *preprocessCore* package available from Bioconductor [11]. Raw signal was background corrected via the normal-exponential convolution model. Multiple probes from a given probe set were summarized using the median polish technique, and between-sample systematic variation was corrected by quantile normalization as implemented in robust multi-array average (RMA) normalization [12,13]. The RMA signal values of multiple probe sets mapping to the same gene were averaged to generate a training-signal matrix using the platform annotation file. This platform annotation file was downloaded using the URL ftp://ftp.ncbi.nlm.nih.gov/geo/platforms/GPLnnn/GPL570/annot/GPL570.annot.gz on January 31, 2014 at 1:27 PM. The descriptors for each of 21,064 genes resulting from this summarization can be found in S2 File. Finally, using the curated annotations, each treatment sample was then compared with its corresponding controls to compute a 21,064 x 31,577 (genes x fold change comparisons) matrix of log_2_ expression fold change values. In other words, fold change values were computed for all 21,064 genes across 31,577 distinct comparisons (experiments where treatment sample is compared against its corresponding controls). The accession details for the corresponding treatment and control sample pairings can be found in S3 File.

To independently evaluate our final sentinel gene set, we later repeated this process on April 17, 2015, downloading all new data available in GEO that was not previously available at the time the original training data was obtained. This process yielded annotations for 13,671 samples from 462 different GEO series. Samples were discarded if no reliable control/treatment pairing could be discerned from the provided data or corresponding CEL files are unavailable. The resulting “independent test dataset” comprises of 11,474 samples, yielding a 21,064 x 4,089 (genes x fold change comparisons) matrix of log_2_ gene expression fold change values. The accession details for the corresponding treatment and control sample pairings can be found in S4 File.

### Gene selection method overview

**Fig 1:**
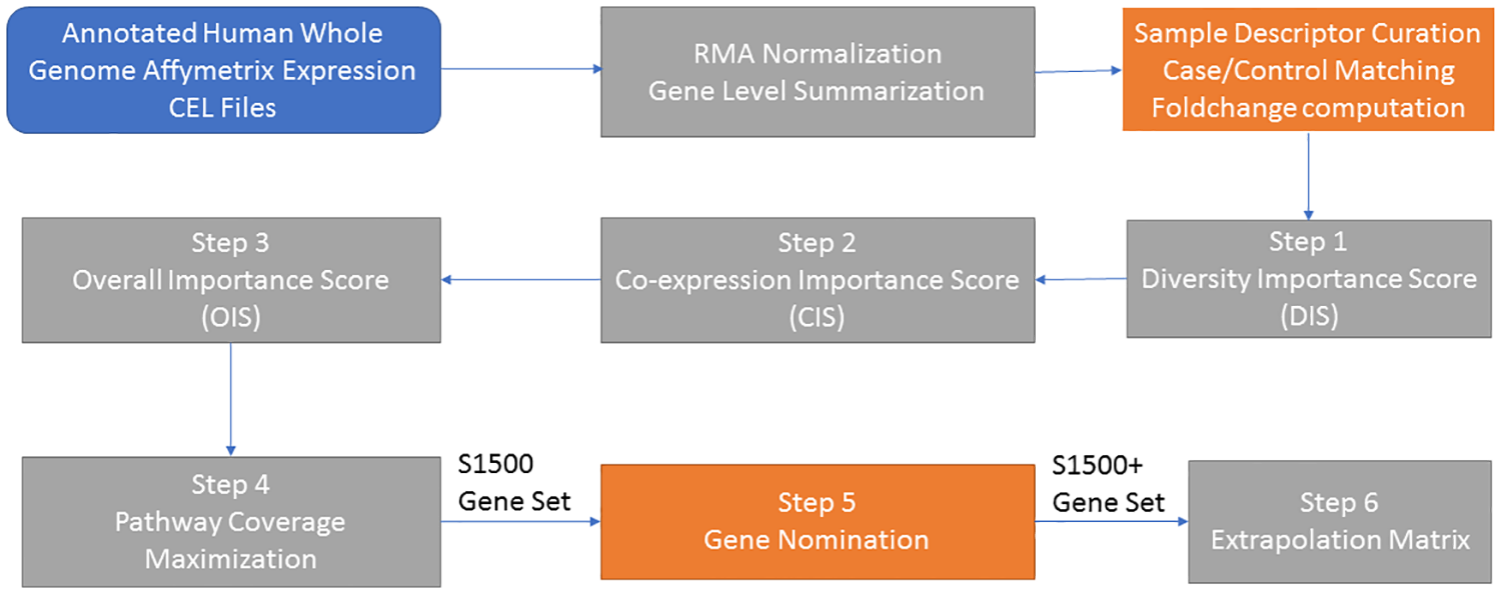
S1500+ gene selection workflow. To compile the S1500+ gene set, a combination of modular data-driven algorithms as well as manual crowd-sourced knowledge-based gene nominations was used to optimize for pathway coverage and the ability to extrapolate to the whole transcriptome.

The strategy for selecting genes was developed to (1) maximally cover the biological response space as represented by gene expression changes; (2) optimally identify key genes that are representative of unique co-expression clusters within the transcriptome; and (3) maximally cover all known and well-annotated biological pathways. Additionally, since we evaluate the sentinel set by its ability to predict the behavior of the non-sentinel genes, it was also critical to develop a corresponding quantitative method to recreate the transcriptional responses of genes not directly included in the sentinel gene list. To select sentinel genes that satisfy the objectives listed above, we have devised a modular, multistep bioinformatics approach (Fig 1). Briefly, log_2_ fold change values were used to compute an Overall Importance Score (OIS). First, each gene was assigned both a Diversity Importance Score (DIS; Step 1) to represent the transcriptional changes caused by all of the diverse experimental treatments represented in these data, and a Co-expression Importance Score (CIS; Step 2) to represent the degree to which this gene was co-expressed with other genes in the transcriptome. Once the DIS and CIS were calculated for each gene, we generated a composite OIS by combining DIS and CIS scores into a single value (Step 3). This overall importance score provided the foundation for a data-driven approach to prioritize genes for inclusion in the sentinel set. To ensure that all major biological pathways were adequately represented in the final sentinel gene list, pathway optimization (Step 4) was performed to ensure that a minimum number of genes from each canonical pathway from Molecular Signature Database (MSigDB v4.0) was included. The resulting S1500 gene set was then augmented using a knowledge-driven selection of additional genes (Step 5) to create the S1500+ gene set. In the final step, we constructed an extrapolation matrix (Step 6) that can be utilized to infer fold changes for the entire transcriptome based solely on expression data measured for the S1500+ subset. Each of these six steps are described in detail below.

### Step 1: Diversity importance score (DIS)

The objective of this step is to assign to each gene a score that represents its contribution toward the biological diversity captured within the gene expression training set. To achieve this, we first clustered the studies from the expression dataset, and then quantified the contributions of individual genes to the primary principal components in each cluster. The DIS score is an average of these contributions for each gene. Under this approach, genes that contribute highly to the primary principal components in many individual clusters will tend to have high DIS values. The DIS score for each gene is calculated using the following procedure:

1. *Perform k-means clustering of experiments (k = 10) using the primary principal components*. Executing k-means clustering using the individual fold change values of all genes across all samples on such a large dataset (~78,000 samples) would be computationally prohibitive. To overcome this hurdle, principal components analysis was performed on the data to identify a reduced set of G “eigengenes.” Here G was determined such that first G eigengenes (principal components) captured 90% of the total variance in the differential gene expression across all experiments. Euclidean distance was used as the dissimilarity metric for clustering.
2. *Compute within-cluster DIS scores for each gene*. Within each resulting cluster, we then recomputed the principal components of the gene expression matrix for the samples included in that cluster. The within-cluster DIS for each gene is defined as a sum of its squared loading coefficients (eigenvector components) for the first *G* principal components.
3. *Summarize cluster-wise DIS for each gene*. Final DIS scores for each gene were computed by summarizing the within-cluster DIS scores using the Tukey bi-weight mean, a robust average that down-weights the contributions of outliers.

### Step 2: Co-expression importance score (CIS)

The co-expression importance score (CIS) assigns a high score to genes that could potentially be used as a “proxy” to represent the expression values for a large number of unmeasured genes. To achieve this, genes were first clustered into sets having similar expression values across samples. Subsequently, a score was given to each gene, with large values assigned to genes that are highly correlated with the remaining genes in the same cluster.

To compute this CIS score, the studies in the expression dataset were randomly partitioned into 20 folds (groups) of equal size. Within each fold, unsupervised hierarchical clustering was performed to identify genes having similar expression profiles. Here, the distance metric used for clustering was one minus the absolute Pearson correlation. Gene clusters were constructed within each fold by cutting the resulting hierarchical clustering at a user-specified distance *h*. A default value of *h* = 0.9 was selected because it resulted in low number of single gene clusters. Within a fold, a particular gene may only belong to a single cluster, and each cluster includes 1 or more genes. Given the resulting clusters, the fold-wise CIS for a gene is defined as the average squared correlation between the expression values of that gene and the other genes within the same cluster (for additional details see Page 2 in S1 File). For clusters with only a single gene, that gene’s CIS score is zero. Finally, as in the case of Step 1a (DIS), the multiple (fold-wise) CIS scores for each gene were summarized using the robust Tukey bi-weight mean.

### Step 3: Overall importance score (OIS)

Once the DIS and CIS scores have been computed for each gene, the two values are then combined into a single Overall Importance Score (OIS). To compute the OIS scores, we first rank-ordered the CIS and DIS scores from smallest to largest, such that a large rank indicates a high score. For a given gene, *g*, the rank of that gene’s CIS score is denoted as rank(CIS(*g*)) and the rank of the gene’s DIS score as rank(DIS(*g*)). The OIS for gene (*g)*, given the total number of genes (*N*), is then defined as:

$$\text{OIS}=\frac{\sqrt{\frac{1}{2}({\text{rank}(\text{CIS}(g))}^{2}+{\text{rank}(\text{DIS}(g))}^{2})}}{N}.$$

OIS scores range between 0 and 1 with large scores indicating “important” genes. To construct the initial S1500 gene set, we chose the 1500 genes having the highest overall importance scores.

### Step 4: Pathway coverage

The OIS score was used to define an initial data-driven list of diverse, representative genes that can serve as a proxy for the remainder of the genome. To ensure that the 1500 chosen genes adequately cover annotated biological pathways, we subsequently used the following procedure to iteratively refine this gene selection.

Canonical pathways from the Molecular Signature Database (MSigDB 4.0) database [14] were downloaded from the Broad Institute (http://software.broadinstitute.org/gsea/msigdb/download_file.jsp?filePath=/resources/msigdb/4.0/msigdb_v4.0_files_to_download_locally.zip). The descriptors for each of the 1,320 canonical pathways are included in S5 File. Then, the gene selection is iteratively refined to increase the pathway coverage until each pathway is represented by at least *q* genes. We used *q* equal to 3 to define the refined S1500 gene set. In each iteration, the selection is updated by replacing the *best exclusion candidate* with the *best inclusion candidate*. The best inclusion candidate is defined as a gene, not currently in the chosen set of 1500 genes, that is contained in the largest number of *uncovered pathways*. An uncovered pathway is any pathway that is not yet represented by at least *q* genes. The best exclusion candidate is defined as a gene in the current selection that overlaps the fewest pathways and has the smallest overall importance score. This procedure is repeated until there are no remaining uncovered pathways. In this way, the initial set of genes is refined using a heuristic procedure that will result in a high-scoring gene set that also contains at least *q* genes from each known biological pathway.

### Step 5: Gene nomination

In addition to the refined S1500 gene set chosen using the data-driven approaches outlined in Steps 1–3, nominated genes were solicited from the public for incorporation into the final gene set. Specifically, the NIEHS/NTP published a Federal Register notice on July 29, 2013 announcing the program goals and requesting nominations for “environmentally responsive genes” to use in evaluating biological consequences of exposure with large numbers of substances via high throughput approaches [15]. In total, over 2,000 unique genes were nominated. Nominated genes were reviewed to eliminate genes that were already included in the gene set after applying the data-driven selection methods from the first three steps. The remaining nominations were then prioritized by a S1500+ working group comprised of 15 Ph.D. level representatives from each of the agencies in the Tox21 partnership, NIEHS/NTP, NCATS, US EPA, and US FDA, with representatives from SciOme. Nominated genes were evaluated for inclusion based on the weight of supporting information provided related to their importance in toxicological and pathological processes. In total, 1,239 additional genes were selected from the nominated set based on their added value. The selected nominations were then combined with the S1500 gene set to create the final S1500+ gene list (version 1) which consists of 2,739 unique genes used in this manuscript for gene selection and extrapolation evaluation. Note that this gene list was recently revised to exclude 2 genes (namely MIR10B and MIR143HG) due to technical difficulties and to include 16 nominated genes that were not present on Affymetrix HG-U133plus2 microarray; resulting in a S1500+ gene list (version 2) consisting of 2753 unique genes.

### Step 6: Extrapolation matrix

The overall goal of selecting the S1500+ gene set was to obtain a small set of genes that can be used to estimate the expression levels of genes that are not directly measured. To achieve this goal, we used principal component regression [16] to compute an extrapolation matrix which can be used to predict the unmeasured portion of the transcriptome using only the measured sentinel genes:

1. Principal component analysis was used to compute the first *K* eigengenes for the transformed sentinel gene fold change matrix. The number of eigengenes to be retained (*K*) was determined by a user-specified parameter (*λ*, in our case 0.01), which is the ratio of the first- and last-selected eigen values.
2. Using this reduced set of eigengenes as explanatory variables and the remaining genes to be extrapolated as dependent variables, standard multivariate regression was used to compute the regression coefficient matrix.
3. Finally, the extrapolation matrix is computed by multiplying the regression coefficient matrix with the original principal component loading coefficient matrix.

The above approach (for additional detail, refer to Page 3 in S1 File) avoids overfitting and, because of the availability of well documented, easy-to-use multithreaded PCA libraries [17,18], is computationally less intensive than alternative approaches, such as regularized or elasticity net regression techniques[19–21], which most reliably operate on one dependent variable at a time.

### Performance evaluation method

1. **Cross validation performance**: We used 20-fold cross validation based on the training dataset described above to evaluate the gene selection method. To accomplish this, the training dataset was randomly divided into 20 different equally sized partitions. Gene selection was then performed 20 times, each time excluding the samples from one partition (20-fold cross validation). In each iteration, sentinel genes were selected from the dataset using both the S1500 method and random selection of 1500 genes. Then, expression measurements for the selected sentinel genes in the excluded partition were used to compute extrapolated fold changes for the remaining unselected genes. These extrapolated values were then compared with the actual, measured fold changes for those genes.
2. **Independent test dataset extrapolation performance**: The entire training dataset was used with the aforementioned gene selection procedure to derive the final S1500+ gene set. The extrapolation performance of this S1500+ gene list was evaluated using newly retrieved data from GEO (independent test dataset) that was not included as part of the original training set used for gene selection. Here, we used the fold change values of sentinel (S1500+) genes from the independent test dataset to generate extrapolated fold change values for remaining genes and compared the extrapolated values with actual fold values.

For both evaluation steps, we performed pathway level performance evaluation using canonical pathways from the MSigDB 4.0. We used the GSEA (generalized Kolomogorov Smirnov test statistic) method [11] to calculate non-normalized pathway enrichment scores (ES) for each pathway. To account for the fact that extrapolation is performed in fold change space, ES computation utilized gene level log2 fold changes instead of conventional signal-to-noise metric. These scores, which range from -1 to 1, indicate the degree to which a given pathway is down- or up-regulated. Finally, the resulting ES based on extrapolated fold change values are compared with ES based on actual fold changes using the same performance statistics previously evaluated at the gene level: Pearson correlation, concordance rate, significance overlap, and mean squared error.

### Functional evaluation of S1500+ gene set: GSE66384 case study

To demonstrate the functional advantage of the data-driven selected S1500 gene set over randomly selected gene lists, we performed a detailed pathway level analysis of the microarray expression data obtained from GEO experiment GSE66384. This experiment is a member of the independent test dataset and was not included in the training data used in the S1500 gene selection. GSE66384 is a study comparing gene expression profiles in T-helper cells from follicular lymphoma versus tonsillectomy [22].

Pathway level enrichment scores were computed to identify differentially expressed pathways in the GSE66384 dataset. We also computed pathway-level Kolomogorov-Smirnov (KS) test significance p-values. This analysis was conducted using the observed GSE66384 expression values for five different subsets of the available genes: (1) the complete microarray dataset; (2) microarray expression values for only the S1500 genes and corresponding extrapolated values; (3) the microarray expression values for a random set of 1500 genes; (4) the microarray expression values for S1500+ genes and corresponding extrapolated values; and (5) the microarray expression values of random gene subset of same size as S1500+ and corresponding extrapolated values.

For each gene subset, we identified differentially expressed pathways by applying an absolute ES threshold of |0.5| and Kolomogorov-Smirnov significance p-value threshold of 0.001. We also computed the recall rate (defined as percent of truly differentially expressed pathways that are correctly detected as differentially expressed) and precision rate (defined as percent of detected differentially expressed pathways that are truly differentially expressed) for each data subset.

## Results

### Gene expression landscape

**Fig 2:**
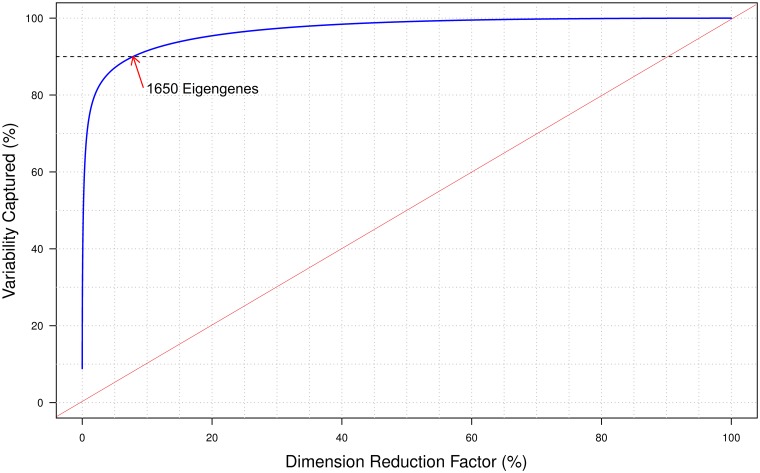
Dimension reduction plot. X-axis shows the percentage of the total principal components (eigengenes) and the Y-axis shows percentage of variability captured. The red line represents the expected relationship given statistically independent gene expression, whereas the blue curve shows the observed relationship.

Due to the highly interconnected, correlated gene expression patterns observed in the human transcriptome, a large percentage of the total variance in gene expression can be captured by a relatively small percentage of genes. For example, as shown in Fig 2, 90% of the total variance observed across all 77,985 experiments in the training set can be captured using less than 8% (1,650/21,064) of the total available eigengenes. In contrast, if the differential expression of all 21,064 genes were statistically independent, we would expect a linear, 1-to-1 relationship (diagonal red line) between the percentage variability captured and dimension reduction factor. Similar findings were previously reported by LINCs consortium ([23], Fig 2) were Connectivity Map based on expression of Landmark 1000 genes was found to reproduce 80% original connections.

**Fig 3:**
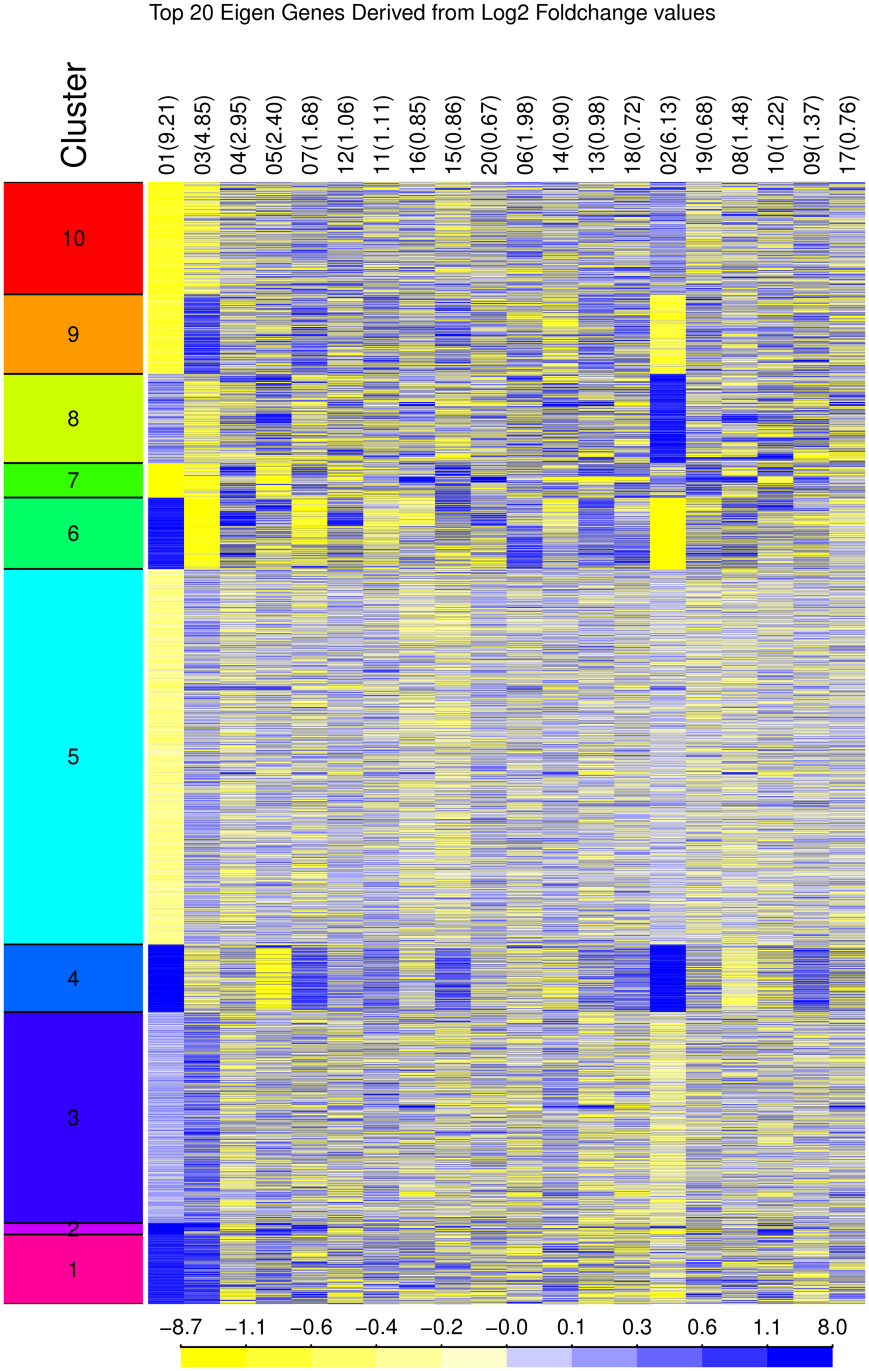
Clustered experiments. K-means clustering (k = 10) was used to cluster experiment data using the first 20 principal components. Fold change values shown are for the top 20 eigengenes. The columns denote principal component indices and percentage of captured variability in parentheses.

The diversity among the expression profiles from this set of experiments is illustrated in Fig 3. The figure displays the results achieved by applying k-means clustering to the experiments as described in Step 1a. Fold change data is displayed for the first 20 principal components, which together capture more than 42% of the variability. Expression patterns are relatively homogenous within clusters, but exhibit larger differences between clusters.

### Selection of sentinel genes

Given the positive results observed when evaluating our data-driven gene selection method on the cross-validated training set as summarized below-, we proceeded to use the full training set to select the S1500 genes. The further review and addition of community-nominated genes resulted in approximately 400 additional high priority genes that were appended to the S1500 gene set along with approximately 800 genes included from the existing Broad L1000 platform. The human S1500+ gene set (2739 genes) selected using this hybrid approach (version 1) was released with a request for public comments through a Federal Register notice on April 15, 2015 (80 FR 20237, April 15, 2015). The resulting revised version 2 of the S1500+ gene set comprises a total of 2,753 genes. The complete S1500+ version 2 gene list can be downloaded here: https://ntp.niehs.nih.gov/results/tox21/researchphases-index.html.

The S1500+ gene set is representative of the highly diverse gene expression changes observed in a comprehensive database of gene expression experiments. In addition, by design, the final inventory of 2,753 genes in the S1500+ covers all major biological pathways available in the MSigDb v4.0. Table 1 shows the average pathway coverage before and after application of Step 2, during which 311 genes were replaced from the initial selection set. Note that among the several alternative gene sets also displayed in the table, only the S1500 and S1500+ sentinel sets cover all 1,320 canonical pathways. In contrast, random gene sets with equivalent sizes cover only 541 (41%) and 852 (65%) of the pathways, respectively.

**Table 1:** Pathway coverage.

	Pathways Covered (total 1320)	Mean Coverage (proportion of Pathway Genes)	Median Coverage (proportion of Pathway Genes)	Mean Multiplicity
**1500 data-driven gene list** **(after Steps 1–3)**	659	0.12	0.08	7.73
**S1500 gene set** **(after Step 1–4)**	1320	0.26	0.25	10.38
**S1500+ (v1)**	1320	0.43	0.43	11.44
**Random 1500**	541 (443, 695)	0.07 (0.05, 0.09)	0.07 (0.05,0.09)	5.88 (5.60, 6.19)
**Random 2739**	852 (759, 946)	0.13 (0.11, 0.15)	0.12 (0.11,0.14)	5.87 (5.66, 6.18)
**L1000** [32]	906	0.17	0.16	12.97

Pathways covered are calculated relative to the 1320 canonical pathways (MSigDB genesets) in MSigDB version 4.0. The pathway level coverage is defined as fraction of genes from pathway that overlap selected gene set. Mean and Median coverage values are derived from pathway level coverage of 1320 canonical pathways from MSigDB (v4.0). The gene level multiplicity metric represents number of pathways a gene is part of. Mean multiplicity is computed using gene level multiplicity metrics across all selected genes. For “Random 1500” and “Random 2739” gene sets parenthesized values represent mean and range (min, max) across 20 alternative randomizations.

### Evaluation of the S1500+ for transcriptome extrapolation

We used 20-fold cross-validation over the training dataset to evaluate the performance of the gene extrapolation method. Table 2 provides a summary of the gene-level extrapolation performance. As displayed in the table, when analyzed at the gene level, similar results were observed for both a random list of 1500 genes and the selected S1500 sentinel genes. However, when the analysis is extended to the pathway level, the results favor the S1500 gene set and selection method over random selection. The high Pearson correlation and concordance rates confirm that the extrapolation algorithm performs well and that the rank order of differential expression is well predicted. At the pathway level, the S1500 selection method leads to improved performance for all four scores in comparison to the random gene selection. For example, the average significance overlap increases from 0.41 to 0.51 when using the S1500 selection method.

**Table 2:** Summary of gene and pathway level extrapolation performance of S1500 gene set on cross-validated training set.

	Pearson Correlation^a^	Concordance Rate^b^	Significance Overlap^c^	Mean Squared Error^d^
**Gene level performance**				
**S1500**	0.79 (0.64, 0.99)	0.94 (0.91, 1.00)	0.34 (0.27, 0.50)	0.22 (0.12, 0.32)
**Random 1500**	0.79 (0.65, 0.99)	0.93 (0.91, 1.00)	0.33 (0.26, 0.51)	0.23 (0.12, 0.31)
**Pathway level performance**				
**S1500**	0.79 (0.44, 0.91)	0.85 (0.55, 0.93)	0.51 (0.43, 0.72)	0.10 (0.02, 0.62)
**Random 1500**	0.75 (0.51, 0.89)	0.82 (0.55, 0.92)	0.41 (0.34, 0.68)	0.12 (0.03, 0.56)

values represent mean and range (min, max) across 20-fold cross validation. Gene-level analyses were conducted using fold change. Pathway-level analyses were conducted on GSEA scores.

^a^ Pearson correlations reflect agreement between extrapolated and measured values

^b^ Concordance rates reflect the agreement between the extrapolated and the measured data calculated as (TP + TN)/(TP+TN+FP+FN)

^c^ Significance overlap relays the proportion of genes/pathways having values (i.e. fold change or GSEA scores) in the top 1% in both the measured and extrapolated datasets

^d^ Mean squared error measures the average squared difference between the extrapolated and measured values

The extrapolation performance of final S1500+ gene set was also evaluated using newly retrieved data (independent test dataset) from GEO that was not included as part of the original training set used for gene selection. The gene level extrapolated signal and pathway level observed and extrapolated enrichment scores for this test dataset are available in S6, S7 and S8 Files, respectively. The results of this evaluation exercise (Table 3) demonstrate that the S1500+ gene set can predict gene expression changes for the unmeasured transcriptome at both the gene and pathway levels. For example, fold changes extrapolated from the S1500+ gene set exhibit Pearson correlations of 0.75 and 0.87 at the gene- and pathway-levels, respectively. Similarly, gene and pathway concordance rates for the S1500+ gene set were 94% and 90%; significance overlaps were 37% and 60%; and the mean squared errors were 0.20 and 0.05 respectively. Note that at the gene level, there is little improvement going from the randomly selected 1500 (or 1500+) genes to the corresponding S1500 (or S1500+) gene sets. Even the addition of extra genes (1500 to 2739) does not significantly improve performance, which is reflected in Fig 2. There is significant improvement at the pathway level however. We see improvements in performance both when adding genes (1500 to 2739) and when intelligently selecting genes (random to S1500).

**Table 3:** Summary of gene and pathway level extrapolation performance of the S1500 and S1500+ gene sets using independent test set.

	Pearson Correlation	Concordance Rate	Significance Overlap	Mean Squared Error
**Gene level Performance**				
**S1500**	0.72	0.93	0.33	0.22
**Random 1500**	0.72 (0.72, 0.73)	0.93 (0.93, 0.93)	0.34 (0.33, 0.34)	0.24 (0.24, 0.25)
**S1500+ (2739 genes)**	0.75	0.94	0.37	0.20
**Random 2739**	0.76 (0.75, 0.76)	0.93 (0.93, 0.93)	0.38 (0.37, 0.38)	0.22 (0.22, 0.22)
**Pathway level performance**				
**S1500**	0.81	0.87	0.52	0.07
**Random 1500**	0.74 (0.73, 0.75)	0.84 (0.84, 0.84)	0.39 (0.37, 0.40)	0.10 (0.09, 0.10)
**S1500+ (2739 genes)**	0.87	0.90	0.60	0.05
**Random 2739**	0.78 (0.77, 0.79)	0.86 (0.86, 0.86)	0.44 (0.42, 0.46)	0.08 (0.08, 0.08)

for the evaluation of random gene lists, a random set of genes was selected 20 times then averaged. The values presented for random gene lists are the mean followed by the minimum and maximum values in brackets.

### Functional evaluation of S1500 gene set: GSE66384 case study

To demonstrate the functional advantage of the data-driven selected S1500 gene set over randomly selected gene lists, we performed a detailed pathway level analysis of the microarray expression data obtained from GEO experiment GSE66384. This experiment is a member of the test set and was not included in the training data used in the S1500 gene selection as mentioned above. GSE66384 is a study comparing gene expression profiles from follicular lymphoma versus tonsillectomy. Pathway analysis was applied to identify the differentially enriched pathways in the GSE66384 dataset (Tables A and B in S9 File). This analysis was conducted using the observed GSE66384 expression values for five different subsets of the available genes: (1) the complete microarray dataset; (2) microarray expression values for only the S1500 genes and corresponding extrapolated values; (3) the microarray expression values for a random set of 1500 genes and corresponding extrapolated values; (4) the microarray expression values for S1500+ genes and corresponding extrapolated values; and (5) the microarray expression values of random gene subset of same size as S1500+ and corresponding extrapolated values.

Most notably, out of the 20 top enriched pathways obtained using the S1500 genes based extrapolated transcriptome, half also occur in the top 20 pathways obtained using the original, complete microarray dataset. Furthermore, all of the top 5 pathways identified from the S1500 genes based extrapolated transcriptome are in the top 20 list from the original microarray dataset. On the other hand, when the random list of 1500 genes is used, only 2 of the resulting top 20 enriched pathways, and none of the top 5 pathways, overlap with the original microarray data-derived enriched pathways. Hence, with regard to enriched pathways derived from extrapolated genes, the agreement with original microarray data was much greater when using the S1500 gene list was used for extrapolation as compared to a random selection of genes.

**Fig 4:**
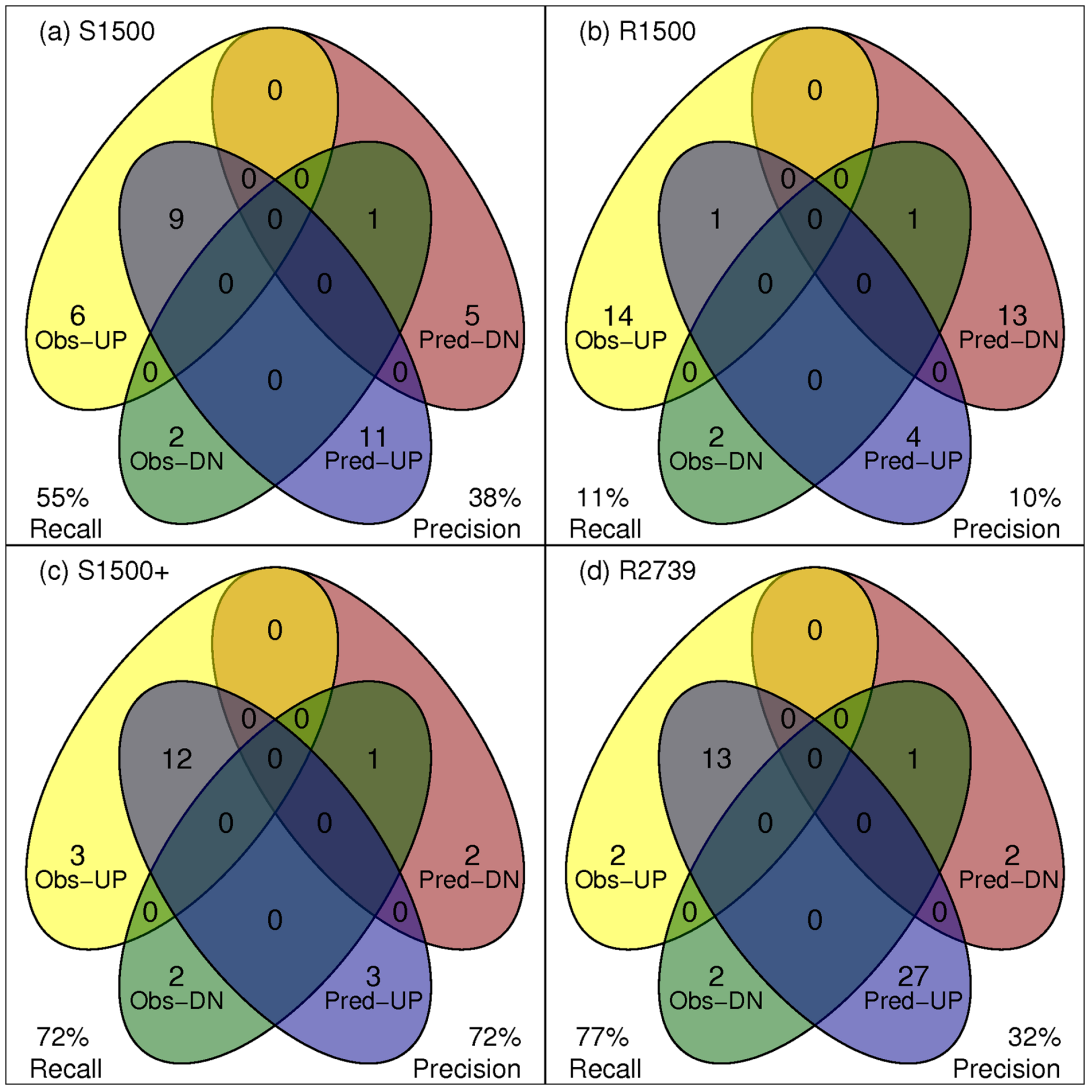
Pathway performance analysis for Follicular Lymphoma vs. Tonsillectomy case study comparison (concordance Venn diagrams). All significantly enriched pathways were identified using enrichment score >0.5 and Kolmogorov Smirnov p-value < 0.001 were included for this analysis. Recall is the percentage of the observed up-/down-regulated genes (Obs-Up and Obs-Down) that were also correctly predicted as up-/down-regulated (Pred-Up/Down). Precision is the percentage of the predicted up- and down-regulated genes that were observed as up- and down-regulated.

The same analysis was repeated for the full S1500+ gene set with similar results. The number of total enriched pathways meeting significance criteria (ES >0.5 and KS p-value <0.001), is summarized in Fig 4. The analysis shows that when using the S1500+ gene set, significant pathways were identified with a recall of 72% (13 out of 18) and a precision of 72% (13 out of 18). When using a randomly selected gene set of the same size, the recall was similar at 77% (14 out of 18), but the precision was lower at 33% (14 out of 43 pathways) with many more pathways incorrectly identified as enriched.

## Discussion

A number of research initiatives have utilized the breadth and reach of transcriptomic signatures for predictive and/or mechanistic purposes. These include endeavors utilizing whole transcriptome microarray platforms for toxicity or drug development research (e.g., DrugMatrix [24], TG-GATEs [25], Broad’s Connectivity Map project [7], NCI-60 cell lines [26]). Together, these studies reveal a clear interconnectedness among gene regulation and expression, reflecting that many of the approximately 20,000 genes in the human genome (and their many isoforms and variants) are not expressed in a completely independent manner. Mechanisms governing gene expression constitute complex phenomena in which groups of genes are regulated in a coordinated fashion under a variety of different cellular states [27]. As a result, expression changes of many genes are tightly synchronized under certain biological contexts. Genes that co-express or are co-regulated are often referred to as “gene modules”, “gene networks” or in some cases “computationally derived pathways” [14]. Transcripts that are regulated in a coordinated manner may encode proteins that we understand to function together biologically and others that we do not yet understand. Nevertheless, the ultimate physiological or pathophysiological status of a biological system is largely the consequence of the interactions between these modules or networks [28–30].

The analyses conducted herein confirm that, in the human transcriptome, the expression of many genes is indeed highly correlated. For example, using only 8% of the possible principal components, we could capture 90% of the total variability observed in a large set of more than 78,000 human microarray experiments. Furthermore, the resulting patterns of gene expression exhibit clearly defined “modules”, discerned by k-means clustering of eigengenes. As a direct result of this high degree of redundancy, it is possible to derive results suggestive of whole-genome expression data using low-cost, high-throughput transcriptomic platforms that focus on the targeted detection of gene expression using a carefully selected subset of the transcriptome. In fact, as we have shown here, even a random selection of 1500 genes can be used to predict the remaining transcriptome with some fidelity.

Our own efforts to select a targeted gene set for use in such platforms build on a similar effort previously conducted by the Broad Institute in the development of their L1000 gene set. The details of the methods used to select the 978 genes included in the L1000 gene set have not yet been published, but the supporting information on their web site indicates that the genes were selected to be “1) minimally redundant, 2) widely expressed in different cellular contexts, and 3) possess inferential value in our statistical models” [31]. The HTT sequencing platforms that we are utilizing have the capacity to include additional genes. For this reason, we have developed a transparent, data-driven procedure that also includes the L1000 gene set with the goal of providing linkage between datasets [32]. By design, our method selects genes that maximally cover the biological response space and favors those genes which optimally identify key genes representative of unique co-expression (co-regulated) clusters. Furthermore, our method also includes a step that is specifically designed to increase pathway coverage. In addition to the purely data-driven approach, we also solicited additional gene nominations from domain experts to create the final S1500+ gene set. In doing so, we have not only ensured that our final S1500+ gene set has excellent performance for all extrapolated genes, but also that those genes that are directly measured are themselves of high relevance to the scientific research community.

Using an independent gene expression test set, we have demonstrated that the S1500+ gene set is capable of accurately predicting fold changes of unmeasured genes in transcriptomics experiments. In addition, by incorporating a pathway coverage heuristic into our method (Step 3), we have doubled the average pathway coverage of our computationally selected gene set in comparison to a random set of 1500 genes. The S1500+ gene set is immediately available for usage in any HTT platforms, and we expect that it will be very useful in a variety of applications. In addition, we are currently applying the same methods described here to independently select a S1500+ gene set for other species (e.g., mouse and rat) for use in animal toxicology studies.

## Conclusions

We have developed a modular bioinformatics approach that utilizes publicly available human transcriptomics data and computes a score for every gene that represents the overall importance of each gene in representing the transcriptional diversity, correlation with other genes based on expression profiling, and known pathway annotation. The S1500 gene set selected with this method provided full coverage of annotated pathways and successfully predicted the gene expression of the remainder of the transcriptome with high accuracy (mean Pearson correlation 0.81, concordance rate 0.87, and significance overlap 0.52) all of which exceeded the performance of a randomly selected gene set of equal size. The addition of community crowd sourced gene nominations to bolster pathological and toxicological relevance and cross-platform comparisons, renders the final S1500+ gene set a robust sentinel gene list amenable to a diversity of applications. The gene selection method presented here can be utilized for selecting gene sets of varying sizes and for generating sentinel gene sets for other species of interest.

## Supporting information

S1 File Sample annotation guideline and additional details. This file provides a detailed description of
data curation process utilizedsteps involved in co-expression importance scoreextrapolation using principal component regression.
(DOCX)

S2 File Gene descriptions. This is a compressed tab delimited file consisting of the following three columns and one row for each gene (21,064) with unique Affymetrix probe set annotation constructed from the GPL570.txt file (Affymetrix Probe Annotation file downloaded from NCBI GEO site on 01/30/2014).
“Gene_Name”–Gene symbols separated by “///” character that mapped to unique collection of Affymetrix Probe Sets.
“Probe Set ID”–Probe Set Identifiers that are mapped to gene symbols separated by “;” character
“Sentinel_Selection_Status”–Binary integer denoting whether gene is “S1500+” sentinel gene set.
(GZ) Click here for additional data file.

S3 File Training data description. This is a compressed tab delimited file which provides a listing of GEO accession identifiers samples that are utilized to produce each of 31,577 differential expression profiles from the training dataset. This file contains the following columns:
“Series”–GEO series identifier
“ID”–Unique identifier for differential expression profile (A vs. B comparison)
“Samples (A)”–GEO accession identifiers of samples that annotated to condition “A” during the curation process (separated by “;” character)
“Samples (B)”–GEO accession identifiers of samples that annotated to condition “B” during the curation process (separated by “;” character).
(GZ) Click here for additional data file.

S4 File Test data description. This is a compressed tab delimited file which provides a listing of GEO accession identifiers for samples that are utilized to produce each of 4,089 differential expression profiles from the test dataset. This file contains the following columns:
“Series”–GEO series identifier
“ID”–Unique identifier for differential expression profile (A vs. B comparison)
“Samples (A)”–GEO accession identifiers of samples that were annotated to condition “A” during the curation process (separated by “;” character)
“Samples (B)”–GEO accession identifiers of samples that were annotated to condition “B” during the curation process (separated by “;” character).
(GZ) Click here for additional data file.

S5 File Pathway description. This is a compressed tab delimited file containing descriptors for each of 1,320 canonical pathways from Broad Institute’s Molecular Signature Database (MSigDB) version 4.0. Note that the following 3 columns were added for convenience
“Symbol.Aliases”—Number of pathway genes with aliases
“Entrez.Exp”—Number entrez gene identfiers mapped to pathway
“Symbols.Exp”—Number of unique gene symbols mapped to pathway.
(GZ) Click here for additional data file.

S6 File Gene level extrapolated signal matrix for test data. This is a compressed tab delimited file consisting of a 18,325 x 4,089 numeric matrix denoting extrapolated log2 fold-change signals for each of 18,325 genes and 4,089 differential expression profiles from the test data set. Note that row-names of this matrix match the “Gene_Name” column from the above gene description file (i.e. S2.txt.gz).
(DOCX)

S7 File Pathway level enrichment score matrix for test data. This is a compressed tab delimited file consisting of a 1,320 x 4,089 numeric matrix denoting pathway enrichment scores for each of 1,320 canonical pathways and 4,089 differential expression profiles from the test data set. Note that row-names of this matrix match the “SYSTEMATIC_NAME” column from above pathway description file (i.e. S5.txt.gz). Also note that these enrichment scores are computed using true log2 fold-change values for all 21,064 genes.
(GZ) Click here for additional data file.

S8 File Pathway level extrapolated enrichment score matrix for test data. This is a compressed tab delimited file consisting of a 1,320 x 4,089 numeric matrix denoting pathway enrichment scores for each of 1,320 canonical pathways and 4,089 differential expression profiles from the test data set. Note that these enrichment score values utilize true observed log2 fold-change values for S1500+ sentinel (2,739) genes and extrapolated signal for other non-sentinel (18,325) genes.
(GZ) Click here for additional data file.

S9 FileSupplementary tables and figures.This file provides following supplementary tablesTable A: GSE66384: Top 20 Pathways for Follicular Lymphoma vs. Tonsillectomy Comparison via S1500 genes and Random 1500 genes based transcriptomesTable B: GSE66384: Top 20 Pathways for Follicular Lymphoma vs. Tonsillectomy Comparison via S1500+ genes and Random 2739 genes based transcriptomeFigure A: Logit-transformed CIS and DIS value density plots(a) Displays empirical density plot of logit transformed Diversity Importance Score (DIS);(b) Displays empirical density plot of logit transformed Co-expression Importance Score (CIS);(c) Displays scatter plots of logit transformed CIS/DIS values, (black points denote top 1500 genes according to overall importance score and gray points denote remaining genes). Note that following logit transformation was used for convenience for creating a plot:
$$\text{logit}\left({\text{x}}^{*}\right)=\text{log}\left({\text{x}}^{*}/\left(1-{\text{x}}^{*}\right)\right){\;\text{where}\;\text{x}}^{*}=\left(\text{x}-\text{min}\left(\text{x}\right)\right)/\left(\text{max}\left(\text{x}\right)-\text{min}\left(\text{x}\right)\right).$$
(DOCX)
