# Supplementary material for: A hybrid gene selection approach to create the S1500+ targeted gene sets for use in high-throughput transcriptomics
Source: PLoS One. 2018 Feb 20;13(2):e0191105. doi: 10.1371/journal.pone.0191105 (PMC5819766; doi:10.1371/journal.pone.0191105)
Supplement: S1 File — This file provides a detailed description of data curation process utilizedsteps involved in co-expression importance scoreextrapolation using principal component regression. (DOCX) [file pone.0191105.s001.docx]

**Supplementary File S1: Sample Annotation Guideline and Additional Details.**

**Sample Annotation Guideline:** For sample from training and test dataset the basic sample description was parsed from MINIML file downloaded from NCBI Gene Expression Omnibus (GEO) repository and converted into easy to process stacked format. Next, a team of trained experts manually deciphered the key sample description attributes to identify key experimental design structures for each GSE series by identifying key primary and secondary/nesting factors such as treatment, dose, exposure duration, organ or cell types, subject identifiers, etc.

Next each sample (GSM accession) from a given GSE series was assigned appropriate character code as follows:

- **Case 1:** Simple study consisting of independent sample sets with t-experimental conditions

Sample Code= ‘*a’* where *a = 0,…,t-1*

In this case experimental condition 1 will be used as baseline to compute

(t-1) fold-change values for subsequent analysis.

- **Case 2:** Study involving same set t-experimental conditions evaluated via m-tissue types

Sample Code*=’a.b’* where *a=0,..,t-1* and *b=1,..m*

In this case (m x (t-1)) fold-change values will be computed by comparing organ-matched comparison by using first experimental condition as baseline.

- **Case 3**: Multi-factor study involving one primary factor (dose) such as subject, chemical exposure, etc. and k-secondary design factors such as chemical exposure duration, dose, tissue, etc.

Sample Code=*’a.b_1_.b_2_…b_k_’* or Sample Code=*’a.d’*

In this case, for each combination of secondary factors, the fold-change comparing various levels of primary factors will be computed for subsequent analysis.

Since same experimental design can lead to slightly different primary/secondary factor detection, in the initial stage of the sample annotation exercise, we conducted a pilot study using a random selection of approximately 5% of the GEO series. The samples from these series were subjected to repeated sample annotation performed by two different curators independently. This pilot study facilitated the identification of case characteristics and provided critical feedback to curation team members.

**Algorithm used for Co-expression Importance Score Computation:**

1. Randomly Partition expression dataset into 20 folds (groups) of equal size.
2. For ***f*** in *1,..,20*
   1. Set aside profiles from fold ***f*** and use remaining samples to compute Pearson correlation for each pair of genes
   2. Perform unsupervised hierarchical clustering of using absolute value of Pearson correlation based distance.
   3. Cut the corresponding dendrogram at user specified height (h=0.9) to identify gene clusters ***G*** as displayed in following figure


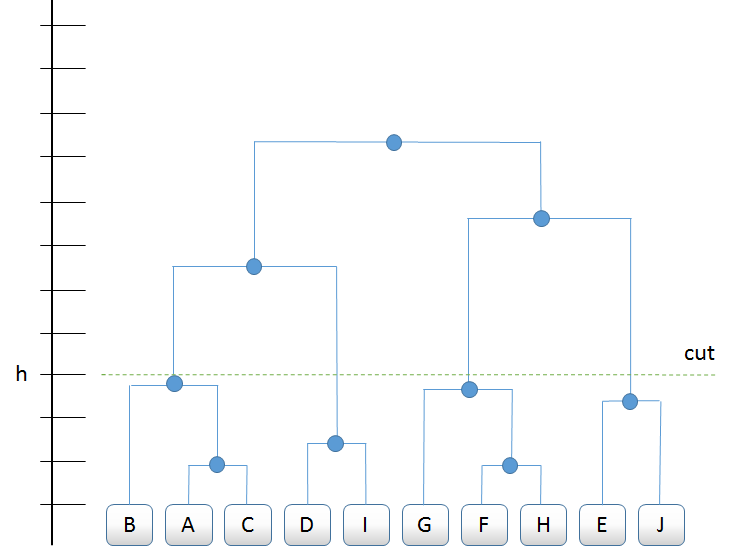


Distance, shown in the vertical axis, is computed as 1 minus the Pearson correlation of expression across samples within fold. In the example above, cutting at *h* results in 4 clusters, with cluster 1 consisting of genes B,A,C. For the CIS, *h*= 0.9.

- 1. for each gene cluster ***c*** in ***G*** ,
     1. compute pairwise Pearson correlation (r_gg’_) for each ***g*** and ***g’***  gene cluster ***c,*** using profiles set aside in step 2-a.
     2. for each gene ***g*** in ***c***, compute fold ***f*** specific *CIS*_gf_ = mean r^2^_gg’_

1. For each gene ***g***, Compute overall CIS using Tukey biweight mean of fold specific *CIS*_gf_

**Principal Component Regression based Extrapolation:**

Let *X* be a training data set consisting of an (*n* x *p*) sentinel gene fold change matrix, where *n* is the number of samples (e.g. ~78,000) and *p* is the number of sentinel genes (~1,500). We assume that *X* has been column-normalized such that the fold changes for each gene have mean 0 and standard deviation of 1.

Denote user-specified dimension reduction factor by λ.

Perform principal component analysis of *X* to compute eigengenes (φ_1_, .., φ_p_) and eigen values (θ_1_, …,θ_p_) identify *k* = min {j: θ_j_/θ_1_≥ λ} and denote the first *k* principal components by a (*p* x *k*) matrix, Ψ = [φ_1_, .., φ_k_],

To perform extrapolation, we first reduce the dimension of the sentinel gene training matrix:

$X^{'}=X$

where the resulting *X’* is an (*n* x *k*) matrix containing the projection of the sentinel gene fold change matrix onto the first *k* principal components.

Then we perform least squares regression using the data in *X’* as explanatory variables and the *r* (e.g. ~20,000) non-sentinel genes from the training data as dependent variables.

Denote the non-sentinel gene fold change training matrix as *Y*, an (*n* x *r*) matrix. Let $\beta$, a (*k* x *r*) matrix, denote the regression coefficients:

$Y=X'\beta$

Now, we can compute an extrapolation matrix as a (*p* x *r*) matrix

$\Delta=\beta$

so that given a new set of measurements, in the form of an (*m* x *p*) matrix $X_{new}$, which contains fold change values for the *p* sentinel genes measured in *m* new samples, we can compute $\hat{Y}$, an (*m* x *r*) matrix, the set of estimated measurements for the *r* unmeasured genes in these *m* samples

$\hat{Y}=X_{new}\Delta$

After performing the extrapolation, $\hat{Y}$ is reversed-normalized using the mean and standard deviations of the corresponding gene-wise fold changes originally observed in the training dataset.
