# Supplementary material for: A hybrid gene selection approach to create the S1500+ targeted gene sets for use in high-throughput transcriptomics
Source: PLoS One. 2018 Feb 20;13(2):e0191105. doi: 10.1371/journal.pone.0191105 (PMC5819766; doi:10.1371/journal.pone.0191105)
Supplement: S6 File — This is a compressed tab delimited file consisting of a 18,325 x 4,089 numeric matrix denoting extrapolated log2 fold-change signals for each of 18,325 genes and 4,089 differential expression profiles from the test data set. Note that row-names of this matrix match the “Gene_Name” column from the above gene description file (i.e. S2.txt.gz). (DOCX) [file pone.0191105.s006.docx]

**Gene Level Extrapolated Signal Matrix for Test Data**

The gene level extrapolated signal matrix file for test data set is currently available at Chemical Effects in Biological Systems (CEBS, <https://tools.niehs.nih.gov/cebs3/ui>) and can be accessed using CEBS Accession number: 002-00050-0001-000-3 or can be accessed directly following URL

<https://tools.niehs.nih.gov/cebs3/views/?action=main.dataReview&bin_id=3812>

The S6 File is a compressed tab delimited file consisting of a 18,325 x 4,089 numeric matrix denoting extrapolated log2 fold-change signals for each of 18,325 genes and 4,089 differential expression profiles from the test data set. Note that row-names of this matrix match the “Gene_Name” column from the above gene description file (i.e. S2.txt.gz).
