# Supplementary material for: A hybrid gene selection approach to create the S1500+ targeted gene sets for use in high-throughput transcriptomics
Source: PLoS One. 2018 Feb 20;13(2):e0191105. doi: 10.1371/journal.pone.0191105 (PMC5819766; doi:10.1371/journal.pone.0191105)
Supplement: S9 File — This file provides following supplementary tables Table A: GSE66384: Top 20 Pathways for Follicular Lymphoma vs. Tonsillectomy Comparison via S1500 genes and Random 1500 genes based transcriptomes Table B: GSE66384: Top 20 Pathways for Follicular Lymphoma vs. Tonsillectomy Comparison via S1500+ genes and Random 2739 genes based transcriptome Figure A: Logit-transformed CIS and DIS value density plots (a) Displays empirical density plot of logit transformed Diversity Importance Score (DIS);(b) Displays empirical density plot of logit transformed Co-expression Importance Score (CIS);(c) Displays scatter plots of logit transformed CIS/DIS values, (black points denote top 1500 genes according to overall importance score and gray points denote remaining genes). Note that following logit transformation was used for convenience for creating a plot: logit(x*)=log(x*/(1−x*))wherex*=(x−min(x))/(max(x)−min(x)). (DOCX) [file pone.0191105.s009.docx]

| **Supplementary Tables and Figures** | | | | | |
| --- | --- | --- | --- | --- | --- |
| **Table A. GSE66384: Top 20 Pathways for Follicular Lymphoma vs. Tonsillectomy Comparison** | | | | | |
| **Microarray measured transcriptome** | | **S1500 genes-based transcriptome** | | **Random 1500 genes-based transcriptome** | |
| **Pathway** | **ES** | **Pathway** | **ES** | **Pathway** | **ES** |
| REACTOME PHOSPHORYLATION OF CD3 AND TCR ZETA CHAINS | 0.918 | **KEGG ASTHMA** | 0.904 | REACTOME STRIATED MUSCLE CONTRACTION | -0.756 |
| KEGG ASTHMA | 0.901 | **KEGG ALLOGRAFT REJECTION** | 0.866 | BIOCARTA CLASSIC PATHWAY | -0.750 |
| KEGG ALLOGRAFT REJECTION | 0.881 | **KEGG GRAFT VERSUS HOST DISEASE** | 0.865 | REACTOME MUSCLE CONTRACTION | -0.732 |
| KEGG TYPE I DIABETES MELLITUS | 0.858 | **KEGG TYPE I DIABETES MELLITUS** | 0.861 | BIOCARTA IL12 PATHWAY | 0.717 |
| KEGG GRAFT VERSUS HOST DISEASE | 0.834 | **KEGG INTESTINAL IMMUNE NETWORK FOR IGA PRODUCTION** | 0.804 | REACTOME SMOOTH MUSCLE CONTRACTION | -0.708 |
| KEGG AUTOIMMUNE THYROID DISEASE | 0.816 | **KEGG ANTIGEN PROCESSING AND PRESENTATION** | 0.767 | **REACTOME INTERFERON ALPHA BETA SIGNALING** | -0.670 |
| KEGG INTESTINAL IMMUNE NETWORK FOR IGA PRODUCTION | 0.813 | KEGG LEISHMANIA INFECTION | 0.763 | PID INTEGRIN1 PATHWAY | -0.643 |
| REACTOME GENERATION OF SECOND MESSENGER MOLECULES | 0.807 | REACTOME ENDOSOMAL VACUOLAR PATHWAY | 0.727 | **REACTOME TRANSPORT OF MATURE TRANSCRIPT TO CYTOPLASM** | 0.628 |
| KEGG ANTIGEN PROCESSING AND PRESENTATION | 0.716 | **REACTOME INTERFERON ALPHA BETA SIGNALING** | -0.711 | REACTOME GLYCOLYSIS | -0.607 |
| REACTOME TCR SIGNALING | 0.712 | **KEGG SYSTEMIC LUPUS ERYTHEMATOSUS** | 0.692 | REACTOME GLUCONEOGENESIS | -0.590 |
| KEGG VIRAL MYOCARDITIS | 0.704 | REACTOME ANTIGEN PRESENTATION FOLDING ASSEMBLY AND PEPTIDE LOADING OF CLASS I MHC | 0.594 | REACTOME ANTIGEN PRESENTATION FOLDING ASSEMBLY AND PEPTIDE LOADING OF CLASS I MHC | 0.563 |
| REACTOME INTERFERON ALPHA BETA SIGNALING | -0.680 | **REACTOME MRNA 3 END PROCESSING** | 0.594 | PID SYNDECAN 1 PATHWAY | -0.562 |
| REACTOME NEF MEDIATES DOWN MODULATION OF CELL SURFACE RECEPTORS BY RECRUITING THEM TO CLATHRIN ADAPTERS | -0.680 | KEGG STEROID BIOSYNTHESIS | -0.587 | REACTOME COLLAGEN FORMATION | -0.560 |
| KEGG SYSTEMIC LUPUS ERYTHEMATOSUS | 0.663 | REACTOME CYTOSOLIC TRNA AMINOACYLATION | 0.568 | KEGG ECM RECEPTOR INTERACTION | -0.558 |
| KEGG CELL ADHESION MOLECULES CAMS | 0.635 | REACTOME DEPOSITION OF NEW CENPA CONTAINING NUCLEOSOMES AT THE CENTROMERE | 0.568 | REACTOME ASSOCIATION OF TRIC CCT WITH TARGET PROTEINS DURING BIOSYNTHESIS | 0.555 |
| REACTOME THE ROLE OF NEF IN HIV1 REPLICATION AND DISEASE PATHOGENESIS | -0.606 | REACTOME PACKAGING OF TELOMERE ENDS | 0.559 | PID AVB3 INTEGRIN PATHWAY | -0.532 |
| REACTOME MRNA 3 END PROCESSING | 0.540 | REACTOME CHOLESTEROL BIOSYNTHESIS | -0.558 | REACTOME EXTRACELLULAR MATRIX ORGANIZATION | -0.530 |
| REACTOME TRANSPORT OF MATURE TRANSCRIPT TO CYTOPLASM | 0.510 | **REACTOME TRANSPORT OF MATURE TRANSCRIPT TO CYTOPLASM** | 0.551 | REACTOME DESTABILIZATION OF MRNA BY AUF1 HNRNP D0 | 0.524 |
| REACTOME CLEAVAGE OF GROWING TRANSCRIPT IN THE TERMINATION REGION | 0.435 | REACTOME TRIGLYCERIDE BIOSYNTHESIS | -0.551 | REACTOME GLUCOSE METABOLISM | -0.512 |
| REACTOME INFLUENZA LIFE CYCLE | 0.391 | KEGG ARGININE AND PROLINE METABOLISM | -0.546 | KEGG PROTEASOME | 0.468 |
| For each transcriptome analysis, Top 20 Canonical pathways from Molecular Signature Database (MSigDB) with Kolomogorov-Smirnov test p-value <=0.001 were ranked by absolute Enrichment Score (ES) value.  The shaded cells of the table denote the pathways that overlap with top-pathway based on the original microarray measured transcriptome. | | | | | |

| **Table B. GSE66384: Top 20 Pathways for Follicular Lymphoma vs. Tonsillectomy Comparison** | | | | | |
| --- | --- | --- | --- | --- | --- |
| **Microarray measured transcriptome** | | **S1500+ genes-based transcriptome** | | **Random 2739 genes-based transcriptome** | |
| **Pathway** | **ES** | **Pathway** | **ES** | **Pathway** | **ES** |
| REACTOME PHOSPHORYLATION OF CD3 AND TCR ZETA CHAINS | 0.918 | **REACTOME PHOSPHORYLATION OF CD3 AND TCR ZETA CHAINS** | 0.926 | **REACTOME PHOSPHORYLATION OF CD3 AND TCR ZETA CHAINS** | 0.871 |
| KEGG ASTHMA | 0.901 | **KEGG ASTHMA** | 0.895 | **KEGG ASTHMA** | 0.853 |
| KEGG ALLOGRAFT REJECTION | 0.881 | **KEGG ALLOGRAFT REJECTION** | 0.871 | **KEGG TYPE I DIABETES MELLITUS** | 0.841 |
| KEGG TYPE I DIABETES MELLITUS | 0.858 | **KEGG TYPE I DIABETES MELLITUS** | 0.869 | **KEGG ALLOGRAFT REJECTION** | 0.828 |
| KEGG GRAFT VERSUS HOST DISEASE | 0.834 | **KEGG GRAFT VERSUS HOST DISEASE** | 0.851 | BIOCARTA BLYMPHOCYTE PATHWAY | 0.827 |
| KEGG AUTOIMMUNE THYROID DISEASE | 0.816 | BIOCARTA BLYMPHOCYTE PATHWAY | 0.846 | BIOCARTA TH1TH2 PATHWAY | 0.819 |
| KEGG INTESTINAL IMMUNE NETWORK FOR IGA PRODUCTION | 0.813 | BIOCARTA INFLAM PATHWAY | 0.791 | **KEGG GRAFT VERSUS HOST DISEASE** | 0.817 |
| REACTOME GENERATION OF SECOND MESSENGER MOLECULES | 0.807 | **KEGG INTESTINAL IMMUNE NETWORK FOR IGA PRODUCTION** | 0.786 | REACTOME ENDOSOMAL VACUOLAR PATHWAY | 0.815 |
| KEGG ANTIGEN PROCESSING AND PRESENTATION | 0.716 | **KEGG AUTOIMMUNE THYROID DISEASE** | 0.776 | **KEGG AUTOIMMUNE THYROID DISEASE** | 0.745 |
| REACTOME TCR SIGNALING | 0.712 | KEGG LEISHMANIA INFECTION | 0.762 | **KEGG INTESTINAL IMMUNE NETWORK FOR IGA PRODUCTION** | 0.738 |
| KEGG VIRAL MYOCARDITIS | 0.704 | **KEGG ANTIGEN PROCESSING AND PRESENTATION** | 0.727 | PID IL12 STAT4PATHWAY | 0.728 |
| REACTOME INTERFERON ALPHA BETA SIGNALING | -0.680 | **REACTOME TCR SIGNALING** | 0.721 | **KEGG ANTIGEN PROCESSING AND PRESENTATION** | 0.728 |
| REACTOME NEF MEDIATES DOWN MODULATION OF CELL SURFACE RECEPTORS BY RECRUITING THEM TO CLATHRIN ADAPTERS | -0.680 | **KEGG VIRAL MYOCARDITIS** | 0.692 | BIOCARTA IL12 PATHWAY | 0.719 |
| KEGG SYSTEMIC LUPUS ERYTHEMATOSUS | 0.663 | **KEGG SYSTEMIC LUPUS ERYTHEMATOSUS** | 0.679 | REACTOME ANTIGEN PRESENTATION FOLDING ASSEMBLY AND PEPTIDE LOADING OF CLASS I MHC | 0.708 |
| KEGG CELL ADHESION MOLECULES CAMS | 0.635 | **REACTOME INTERFERON ALPHA BETA SIGNALING** | -0.641 | REACTOME CHONDROITIN SULFATE BIOSYNTHESIS | -0.700 |
| REACTOME THE ROLE OF NEF IN HIV1 REPLICATION AND DISEASE PATHOGENESIS | -0.606 | REACTOME REGULATION OF IFNA SIGNALING | -0.629 | KEGG LEISHMANIA INFECTION | 0.697 |
| REACTOME MRNA 3 END PROCESSING | 0.540 | REACTOME CHOLESTEROL BIOSYNTHESIS | -0.615 | PID NFAT TFPATHWAY | 0.692 |
| REACTOME TRANSPORT OF MATURE TRANSCRIPT TO CYTOPLASM | 0.510 | **KEGG CELL ADHESION MOLECULES CAMS** | 0.605 | KEGG VIRAL MYOCARDITIS | 0.686 |
| REACTOME CLEAVAGE OF GROWING TRANSCRIPT IN THE TERMINATION REGION | 0.435 | REACTOME DEPOSITION OF NEW CENPA CONTAINING NUCLEOSOMES AT THE CENTROMERE | 0.496 | REACTOME NEP NS2 INTERACTS WITH THE CELLULAR EXPORT MACHINERY | 0.644 |
| REACTOME INFLUENZA LIFE CYCLE | 0.391 | **REACTOME MRNA 3 END PROCESSING** | 0.474 | REACTOME DESTABILIZATION OF MRNA BY AUF1 HNRNP D0 | 0.642 |
| For each transcriptome analysis, Top 20 Canonical pathways from Molecular Signature Database (MSigDB) with Kolomogorov-Smirnov test p-value <=0.001 were ranked by absolute Enrichment Score (ES) value.  The shaded cells of the table denote the pathways that overlap with top-pathway based on the original microarray measured transcriptome. | | | | | |

**Figure A. Logit-transformed CIS/DIS Density and Correlation Plot**


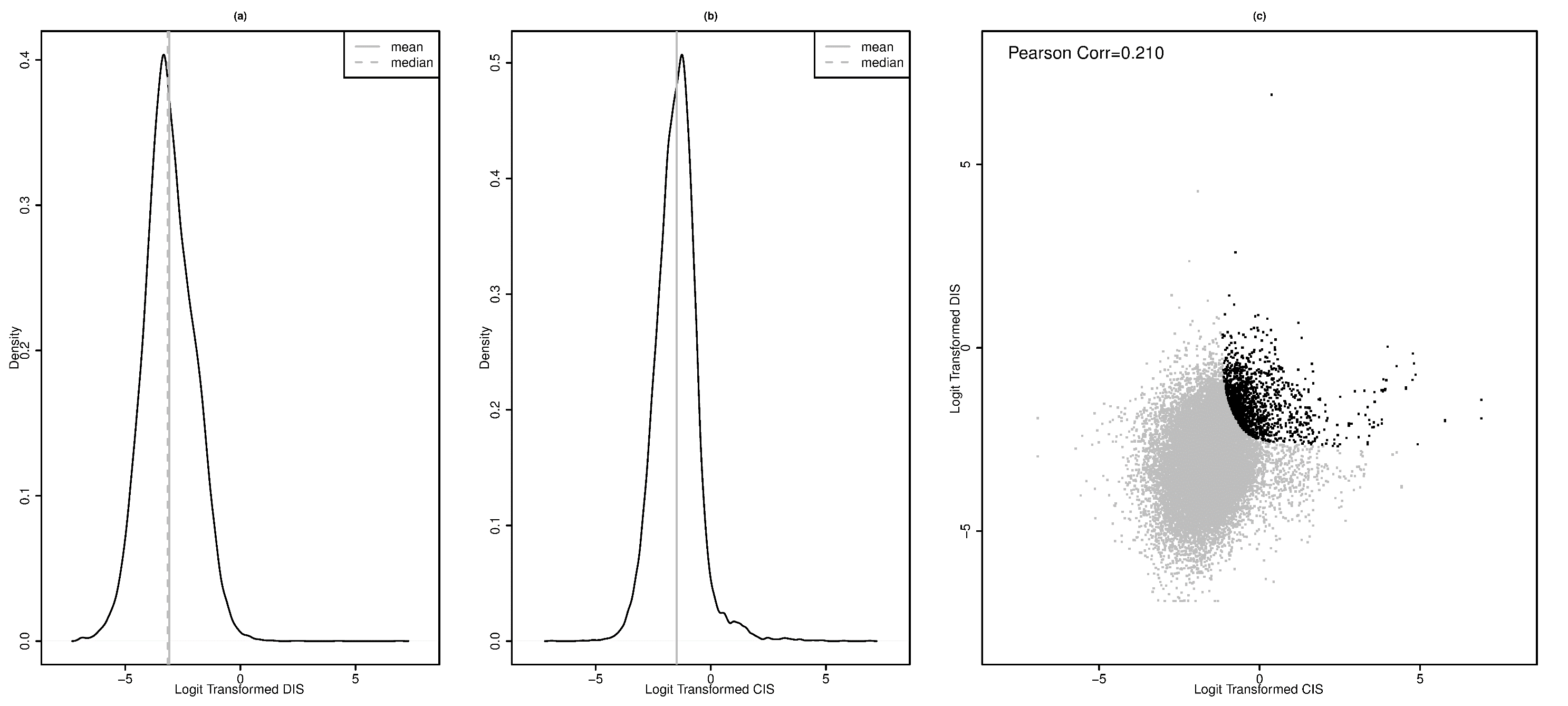


1. Displays empirical density plot of logit transformed Diversity Importance Score (DIS); (b) Displays empirical density plot of logit transformed Co-expression Importance Score (CIS); (c) Displays scatter plots of logit transformed CIS/DIS values, (black points denote genes top 1500 genes according to overall importance score and gray points denote remaining genes). Note that following logit transformation was used plot convenience

logit(x^*^) = log(x^*^/(1- x^*^)) where x^*^=(x-min(x))/(max(x)-min(x))
